# Multivessel vs. Culprit Vessel-Only Percutaneous Coronary Intervention for ST-Segment Elevation Myocardial Infarction in Patients With Cardiogenic Shock: An Updated Systematic Review and Meta-Analysis

**DOI:** 10.3389/fcvm.2022.735636

**Published:** 2022-04-15

**Authors:** Bingquan Xiong, Huiping Yang, Wenlong Yu, Yunjie Zeng, Yue Han, Qiang She

**Affiliations:** Department of Cardiology, The Second Affiliated Hospital of Chongqing Medical University, Chongqing, China

**Keywords:** myocardial infarction, cardiogenic shock, percutaneous coronary intervention, meta-analysis, clinical outcomes

## Abstract

**Background:**

The optimal revascularization strategy in patients with ST-segment elevation myocardial infarction (STEMI) complicating by cardiogenic shock (CS) remains controversial. This study aims to evaluate the clinical outcomes of multivessel percutaneous coronary intervention (MV-PCI) compared to culprit vessel-only PCI (CO-PCI) for the treatment, only in patients with STEMI with CS.

**Methods:**

A comprehensive literature search was conducted. Studies assessed the efficacy outcomes of short (in-hospital or 30 days)/long-term mortality, cardiac death, myocardial reinfarction, repeat revascularization, and safety outcomes of stroke, bleeding, acute renal failure with MV-PCI vs. CO-PCI in patients with STEMI with CS were included. The publication bias and sensitivity analysis were also performed.

**Results:**

A total of 15 studies were included in this meta-analysis. There was no significant difference in short- and long-term mortality in patients treated with MV-PCI compared to CO-PCI group [odds ratio (OR) = 1.17; 95% confidence interval (CI), 0.92–1.48; OR = 0.86; 95% CI, 0.58–1.28]. Similarly, there were no significant differences in cardiac death (OR = 0.67; 95% CI, 0.44–1.00), myocardial reinfarction (OR = 1.24; 95% CI, 0.77–2.00), repeat revascularization (OR = 0.75; 95% CI, 0.40–1.42), bleeding (OR = 1.53; 95% CI, 0.53–4.43), or stroke (OR = 1.42; 95% CI, 0.90–2.23) between the two groups. There was a higher risk in acute renal failure (OR = 1.33; 95% CI, 1.04–1.69) in patients treated with MV-PCI when compared with CO-PCI.

**Conclusion:**

This meta-analysis suggests that there may be no significant benefit for patients with STEMI complicating CS treated with MV-PCI compared with CO-PCI, and patients are at increased risk of developing acute renal failure after MV-PCI intervention.

## Introduction

Cardiogenic shock (CS) occurs in 6–12% of patients with ST-segment elevation myocardial infarction (STEMI) and is associated with increased mortality (1–3). Previous randomized study has suggested that early revascularization in patients with acute myocardial infarction (AMI) with CS could improve the short/long-term survival rate (4). However, there remains high mortality at 40–50% despite the increasing use of early revascularization with percutaneous coronary intervention (PCI) among these patients (2). It was reported that most patients with STEMI with CS have underlying multivessel coronary artery disease (CAD), which is associated with worse outcomes (5–7). Previous randomized controlled trials (RCTs) have suggested that multivessel PCI (MV-PCI) is associated with improved clinical outcomes compared with culprit-vessel only PCI (CO-PCI), but these trials did not comprise patients with CS (8–11). Meanwhile, limited randomized data exist regarding the treatment effect of MV-PCI compared with CO-PCI for patients with STEMI with CS. The United States guidelines suggest that due to pump failure, for patients with STEMI with CS, PCI of a severe stenosis in a large non-infarct artery might improve hemodynamic stability and should be considered during the primary procedure (12). Similarly, the European guidelines on myocardial revascularization state that during STEMI, MV-PCI should be considered in patients with CS in the presence of multiple, critical stenosis or highly unstable lesions, and whether there is persistent ischemia after PCI on the supposed culprit lesion remains unclear (13).

However, the supporting evidence is largely based on pathophysiology considerations and extrapolation of data from clinical trials that included patients with hemodynamically stable STEMI, but not on non-randomized studies in patients with CS. Observational studies have revealed conflicting results when comparing MV-PCI vs. CO-PCI in AMI patients with CS (1, 14–26). In addition, several observational studies have also included patients with STEMI and non-STEMI (NSTEMI), which have different clinical profile, treatment, and outcomes (1, 15, 17, 18, 24). Furthermore, prior meta-analyses have included studies in patients with or without shock when comparing the clinical outcomes of MV-PCI vs. CO-PCI (27, 28). Data for patients with STEMI with CS alone may still be inadequate.

Therefore, we reconducted a systematic review and meta-analysis to investigate the clinical outcomes of MV-PCI compared to CO-PCI for the treatment only in patients with STEMI with CS.

## Methods

### Data Sources and Search Strategy

Two authors (Bingquan Xiong and Qiang She) systematically searched the databases of PubMed, Web of Science, and Medicine for related articles published in English language before December 1, 2020. We used the following keywords: “percutaneous coronary intervention,” “PCI,” “ST-segment elevation myocardial infarction,” “STEMI,” “myocardial infarction,” “cardiogenic shock,” and “multivessel disease” for the search. We included the studies that met the following criteria: (1) studies included patients presented with STEMI and CS, (2) studies comparing the clinical outcomes of MV-PCI vs. CO-PCI, (3) studies that included more than 10 cases in each treatment group, and (4) studies where, at minimum, reported data of interest on short-term mortality for each group. We excluded studies that met any one of the following criteria: (1) review articles, (2) duplicate publication, and (3) abstract or conference publications. In addition, the reference lists of retrieved articles were manually searched for potentially relevant articles. Any difference in article assessments between the two authors was resolved by group discussion. The present study was performed based on the Preferred Reporting Items for Systematic Reviews and Meta-Analyses (PRISMA) statement (29).

### Data Extraction and Quality Assessment

Two authors (Bingquan Xiong and Qiang She) independently extracted the data using a standardized approach, and disagreements were resolved by consensus. Data were collected as follows: study and patient characteristics, baseline clinical characteristics, interventional details, data of clinical outcomes, and follow-up duration. We assessed the quality of included studies using the Newcastle–Ottawa scale (NOS), which assigns a star to each item based on the three domains: selection of study groups (4 criteria), comparability of groups (1 criterion), and ascertainment of outcome/exposure (3 criteria) (30).

### Definition and Endpoints

The CO-PCI was defined as PCI confined to culprit vessel lesions only. The MV-PCI was defined as PCI confined to the culprit vessel lesions and ≥ 1 non-culprit vessel lesions. The number of additional PCI received was comparable between the two groups. Multivessel disease (MVD) was defined as the angiographic detection of significant stenosis (≥50% of lumen diameter) in at least one major non-infarct-related artery. As for the follow-up time, the short term was defined as the time period until hospital discharge or 30 days following the index hospitalization, whereas the long term was defined as the time period extending ≥6 months after index hospitalization. The interest primary outcome was short-term all-cause mortality in this study. Secondary efficacy outcomes included long-term mortality, cardiac death, myocardial reinfarction, and repeat revascularization. Secondary safety outcomes were stroke, bleeding, and renal failure. Since the follow-up time of short term and long term was different across studies, we used the longest available follow-up data from each study for the outcomes of interest in our analysis.

### Statistical Analysis

All the extracted data were pooled to estimate the combined odds ratio (OR) and 95% confidence interval (CI) using the random or fixed-effects models, based on whether there is the existence of heterogeneity. We conducted the sensitivity analysis by recalculating the combined effect estimates after omitting one study at a time (leave-one-out method). The presence of heterogeneity was assessed using *I*^2^ statistics and the Cochrane Q tests, when values of *I*^2^ > 50% and *p* ≤ 0.1 for the Cochrane Q test were considered as the existence of substantial heterogeneity. Publication bias was assessed using Egger’s regression test and visual inspection of asymmetry in funnel plots. All the statistical tests were two-tailed, and *p* < 0.05 was considered as statistically significant. We conducted all the analyses using the STATA version 14 (StataCorp LP, College Station, TX, United States).

## Results

### Characteristics of the Included Studies

**FIGURE 1:**
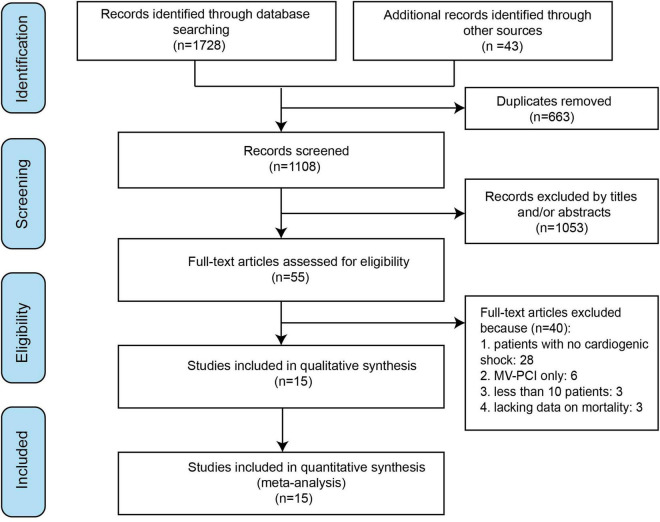
Flowchart for the systematic literature search.

A total of 15 studies were finally included in the meta-analysis (1, 14–26, 31). The detailed study selection process is shown in Figure 1. Table 1 summarizes the characteristics of the included individual studies. Among the 15 studies, 1 was an RCT, 1 was a *post hoc* analysis of RCT, 6 were retrospective, and 7 were prospective. Of the 13 non-randomized studies, 2 studies were from single center, whereas the remaining studies were from national multicenter registries. The definitions of MVD and CS were somewhat different. A total of 4 patients in CO-PCI group received coronary artery bypass graft (CABG) (23), whereas 6 patients in MV-PCI group received scheduled non-urgent CABG (20). Three and 1 patients in MV-PCI group received repeat PCI on target vessel and on non-target vessel, respectively. And 9 patients in CO-PCI group received repeat PCI on non-target vessel (23). However, there was no difference in the incidence rate of repeated PCI and CABG after routine PCI treatment between the two groups (23). The baseline characteristics of patient for the treatment groups of MV-PCI and CO-PCI are comparable and are summarized in Table 2.

**TABLE 1:** Summary characteristics of included studies in the meta-analysis.

Authors (Year)	Study design	Setting	Number of patients	Clinical symptom	PCI strategies subgroups n (%)		Follow-up (months)	Exclusion criteria	Endpoints
Thiele et al. (31)	RCT	Multicenter	706	MI complicated by CS with MVD	342 (48.3)	344 (48.7)	12	Resuscitation >30 min; no intrinsic heart action; severe cerebral deficit; primary urgent CABG; single-vessel coronary artery disease; mechanical cause of CS; onset of shock >12 h; age >90 years; shock with a non-cardiogenic cause; massive pulmonary embolism; other severe concomitant disease with life expectancy <6 months	All-cause mortality, renal replacement therapy, bleeding, and stroke
Cavender et al. (16)	Prospective, observational	Single center	199	STEMI complicated by CS with MVD	43 (22)	156 (78)	31.2	SV disease; no evidence of CS; definite indications for surgery	All-cause mortality assessed at 30 days and the median follow-up of 2.6 years
Yang et al. (23)	Prospective, observational	Multicenter	338	STEMI complicated by CS with MVD	60 (17.8)	278 (82.2)	Median 7.5	Treatment with strategies other than primary PCI; mechanical complications; and left main coronary artery disease	Primary: all-cause mortality assessed at a median follow-up of 224 days; Secondary: in-hospital mortality and MACEs
Jeger et al. (19)	Prospective, observational	Multicenter	1909	STEMI complicated by MVD	442 (23.2)	1467 (76.8)	Median 12.6	NA	Primary: 1 year all-cause mortality; Secondary: MACCEs
Park et al. (21)	Prospective, observational	Multicenter	510	STEMI complicated by CS with MVD	124 (24.3)	386 (75.7)	Median 6.5	Missing initial vital signs information and a NSTEMI final diagnosis	All-cause mortality, cardiac death, MI, revascularization, MACE
Zeymer et al. (24)	*Post hoc* analysis of RCT	Multicenter	451	STEMI and NSTEMI complicated by CS	167 (37)	284 (63)	12	Resuscitation >30 min; severe cerebral deficit; mechanical causes of CS; onset of shock >12 h; shock of other cause; severe peripheral artery disease precluding IABP insertion or severe aortic regurgitation; age >90 years, other severe concomitant disease with life expectancy <6 months	Primary: 30-day all-cause mortality; Secondary: 6-and 12-month mortality, reinfarction, renal replacement therapy, GUSTO moderate, severe, or life-threatening bleeding
Bauer et al. (15)	Retrospective, observational	Multicenter	336	MI complicated by CS with MVD	82 (24)	254 (76)	In-hospital	LM; prior CABG; only 1 ≥ 70% stenosed vessel	All-cause mortality
van der Schaaf et al. (22)	Retrospective, observational	Single center	161	STEMI complicated by CS (single vessel disease, MVD without CTO, and CTO)	37 (23)	124 (77)	12	NA	All-cause mortality
Zeymer et al. (1)	Retrospective, observational	Multicenter	735	MI (STEMI/NSTEMI) complicated by CS with MVD	173 (23.5)	562 (76.5)	In-hospital	LM; prior CABG	All-cause mortality, non-fatal MI, stroke, bleeding, dialysis
Lee et al. (25)	Prospective, observational	Multicenter	659	STEMI and CS	260 (39.5)	399 (60.5)	12	NSTEMI, >12 h onset, no CS, underwent thrombolytic, single vessel disease, failed or suboptimal PCI of IRA, loss of follow-up before 1 year	1 year all cause death, POCO (a composite of all-cause death, any MI, and any repeat revascularization)
McNeice et al. (26)	Retrospective, observational	Multicenter	696	AMI and CS	235 (33.7)	414 (59.3)	12	LM, indication for surgery	Mortality at 30 days and 1 year
Cavender et al. (14)	Retrospective, observational	Multicenter	3,087	STEMI complicated by MVD	433 (14)	2,654 (86)	In-hospital	PCI of left main disease, staged MV-PCI before hospital discharge, thrombolytic	All-cause mortality, stroke, renal failure, bleeding
Mylotte et al. (20)	Prospective, observational	Multicenter	169	STEMI complicated by CS and resuscitated CA	66 (39)	103 (61)	6	Late presentation (>24 h), staged MV-PCI	All-cause mortality, death because of CS, recurrent cardiac arrest, composite of these end points
Jaguszewski et al. (18)	Retrospective, observational	Multicenter	243	STEMI	85 (35)	158 (65)	In-hospital	NA	MACE, all-cause mortality, MI, stroke
Hambraeus et al. (17)	Prospective, observational	Multicenter	330	MVD	67 (60.3)	263 (79.7)	12	Single-vessel disease, prior CABG	Composite of all-cause death, MI, repeat revascularization

*STEMI, ST-segment elevation myocardial infarction; NSTEM; none-ST-segment elevation myocardial infarction; CS, cardiogenic shock; MVD, multivessel disease; RCT, randomized controlled trial; MI, myocardial infarction; AMI, acute myocardial infarction; MACEs, major adverse cardiac events; SV, single vessel; PCI, percutaneous coronary intervention; MV-PCI, multivessel percutaneous coronary intervention; GUSTO, Global Utilization of Streptokinase and Tissue Plasminogen Activator for Occluded Coronary Arteries trial; NA; not available; IRA, infarct related artery; CTO, chronic total occlusion; POCO, patient oriented composite outcomes; MACCEs, major adverse cardiovascular and cerebrovascular events; CABG, coronary artery bypass grafting; CA, cardiac arrest.*

**TABLE 2:** Baseline characteristics according to treatment strategy reported in the individual studies.

Variable	Cavender et al. (16)			Yang et al. (23)			Hambraeus et al. (17)			Jeger et al. (19)			Lee et al. (25)		
					
	MV-PCI	CO-PCI	P	MV-PCI	CO-PCI	P	MV-PCI	CO-PCI	P	MV-PCI	CO-PCI	P	MV-PCI	CO-PCI	P
Age, years	63 ± 14	66 ± 13	0.27	67	70	0.062	68.2 ± 11.8	71.3 ± 10.9	NA	63.3 ± 11.6	65.0 ± 11.7	0.05	66.2 ± 12.4	67.3 ± 12.8	0.27
				(55.3-75.0)	(60.0-78.0)										
Male gender,%	72	62	0.2	63.3	57.9	0.439	67.2	65.4	NA	78.7	77.9	0.74	73.5	74.9	0.67
BMI, kg/m^2^	29 ± 6	28 ± 6	0.55	NA	NA	NA	NA	NA	NA	NA	NA	NA	23.6 ± 3.1	23.4 ± 3.2	0.4
**Cardiovascular risk factors,%**															
Smoking	67	71	0.7	40	35.6	0.522	49.3	41.9	NA	39.7	39.1	0.86	40.4	36.3	0.3
Hypertension	72	79	0.3	50	57.9	0.262	38.8	39.5	NA	58.6	61.3	0.34	52.3	54.6	0.56
Hyperlipidemia	16	24	0.26	21.7	23.4	0.775	22.4	16.7	NA	53.4	55.9	0.39	46.9	46.6	0.94
DM	35	31	0.61	21.7	16.5	0.343	26.9	23.6	NA	18.5	17.1	0.51	41.2	40.9	0.94
Prior MI	44	31	0.1	8.3	4.7	0.336	NA	NA	NA	NA	NA	0.92	6.5	9	0.25
Prior PCI	7	6.4	0.89	3.3	6.1	0.545	NA	NA	NA	NA	NA	0.28	NA	NA	NA
Prior CABG	12	11	0.89	0	1.4	0.999	NA	NA	NA	NA	NA	NA	NA	NA	NA
Heat failure	NA	NA	NA	NA	NA	NA	NA	NA	NA	1.2	1.5	0.82	0.8	3.3	0.04
Stroke	NA	NA	NA	NA	NA	NA	NA	NA	NA	NA	NA	NA	NA	NA	NA
Peripheral artery disease	14	15	0.82	NA	NA	NA	NA	NA	NA	NA	NA	0.47	NA	NA	NA
Chronic kidney disease	19	10	0.14	NA	NA	NA	NA	NA	NA	4.2	3.7	0.67	33.5	39.3	0.13
Cardiac arrest	NA	NA	NA	NA	NA	NA	NA	NA	NA	4.3	2.9	0.16	32.7	37.8	0.18
**Hemodynamics and functional parameters**															
Mean heart rate, beats/min	94 ± 27	85 ± 21	0.06	71.8 ± 35.2	66.5 ± 32.7	0.264	NA	NA	NA	NA	NA	NA	NA	NA	NA
SBP, mmHg	106 ± 23	107 ± 26	0.96	87.6 ± 33.8	83.0 ± 39.0	0.402	NA	NA	NA	NA	NA	NA	NA	NA	NA
LV ejection fraction	24 ± 9	3 ± 14	0.01	48.5 ± 15.3	45.9 ± 13.9	0.257	NA	NA	NA	NA	NA	NA	44.3 ± 13.2	47 ± 12.7	0.01
**Angiographic parameters,%**															
Three-vessel disease	51	52	0.75	46.7	44.2	0.732	25.4	51.3	NA	NA	NA	0.06	33.8	33.3	0.89
TIMI-flow 3 post-PCI	NA	NA	NA	80	84.2	0.43	NA	NA	NA	NA	NA	NA	NA	NA	NA

**Variable**	**Park et al. (21)**			**McNeice et al. (26)**			**Zeymer et al. (24)**			**Bauer et al. (15)**			**van der Schaaf et al. (22)**		
					
	**MV-PCI**	**CO-PCI**	**P**	**MV-PCI**	**CO-PCI**	**P**	**MV-PCI**	**CO-PCI**	**P**	**MV-PCI**	**CO-PCI**	**P**	**MV-PCI**	**CO-PCI**	**P**

Age, years	65.5 (55.0-75.0)	68.0 (57.0-76.0)	0.176	NA	NA	NA	69 ± 12	68 ± 12	0.55	67.2 ± 12.2	65.4 ± 12.2	0.22	NA	NA	NA
Male gender,%	71	65.8	0.287	75.3	75.4	0.99	74	70	0.42	71	68	0.61	NA	NA	NA
BMI, kg/m^2^	24.0 (22.0-26.0)	23.0 (21.0-26.0)	0.343	NA	NA	NA	NA	NA	NA	27.1 ± 4.3	27.6 ± 4.4	0.57	NA	NA	NA
**Cardiovascular risk factors,%**															
Smoking	47.6	46.6	0.837	19.1	27.4	0.98	28	36	0.09	55	54	0.94	NA	NA	NA
Hypertension	53.7	54.5	0.888	59.5	58.6	0.88	68	75	0.08	60	67	0.27	NA	NA	NA
Hyperlipidemia	9.8	9.7	0.969	46.5	41.6	0.35	42	40	0.59	47	55	0.3	NA	NA	NA
DM	25.6	23.3	0.599	34.6	29.9	0.62	40	32	0.1	40	35	0.51	NA	NA	NA
Prior MI	NA	NA	NA	26	25.8	0.98	18	28.9	0.01	32	36	0.45	NA	NA	NA
Prior PCI	NA	NA	NA	22.6	25.1	0.46	13.9	26.4	0	9	13	0.38	NA	NA	NA
Prior CABG	NA	NA	NA	NA	NA	NA	4.8	6.7	0.41	NA	NA	NA	NA	NA	NA
Heat failure	NA	NA	NA	NA	NA	NA	NA	NA	NA	9	11	0.69	NA	NA	NA
Stroke	NA	NA	NA	NA	NA	NA	11.4	7.4	0.15	8	8	0.98	NA	NA	NA
Peripheral artery disease	NA	NA	NA	NA	NA	NA	NA	NA	NA	7	9	0.5	NA	NA	NA
Chronic kidney disease	NA	NA	NA	25.2	15.5	0.006	19.9	24.3	0.28	9	6	0.35	NA	NA	NA
Cardiac arrest	NA	NA	NA	NA	NA	NA	NA	NA	NA	NA	NA	NA	NA	NA	NA
**Hemodynamics and functional parameters**															
Mean heart rate, beats/min	66.0 (50.0-81.0)	62.0 (48.0-80.0)	0.426	NA	NA	NA	96 ± 27	90 ± 26	0.04	NA	NA	NA	NA	NA	NA
SBP, mmHg	80.0 (73.0-90.0)	80.0 (70.0-90.0)	0.282	NA	NA	NA	97 ± 22	92 ± 23	0.02	NA	NA	NA	NA	NA	NA
LV ejection fraction	50.0 (39.0-60.0)	50.0 (43.0-58.0)	0.917	30.9	29.3	0.76	NA	NA	NA	NA	NA	NA	NA	NA	NA
**Angiographic parameters,%**															
Three-vessel disease	46	39.9	0.315	NA	NA	NA	73	62	0.02	51	46	0.42	NA	NA	NA
TIMI-flow 3 post-PCI	90.7	88	0.734	NA	NA	NA	83	80	0.53	77	71	0.34	NA	NA	NA

**Variable**	**Zeymer et al. (1)**			**Thiele et al. (31)**			**Cavender et al. (14)**			**Mylotte et al. (20)**			**Jaguszewski et al. (18)**		
					
	**MV-PCI**	**CO-PCI**	**P**	**MV-PCI**	**CO-PCI**	**P**	**MV-PCI**	**CO-PCI**	**P**	**MV-PCI**	**CO-PCI**	**P**	**MV-PCI**	**CO-PCI**	**P**

Age, years	68	70.2	0.2	70	70	NA	60	62	< 0.01	65.0 ± 12.4	68.5 ± 11.8	0.088	64.7 ± 11.7	65 ± 11.2	NA
				(60-77)	(60-78)		(52-72)	(53-73)							
Male gender,%	72.3	70.8	0.7	78.1	74.9	NA	71.5	72.1	0.32	66	71.8	0.598	77.6	74.7	NA
BMI, kg/m^2^	NA	NA	NA	27	27	NA	28.1 (25.0-31.6)	27.7	< 0.01	25	26	0.288	NA	NA	NA
				(25-29)	(24-29)			(24.8-31.3)		(23.3-27.0)	(23.0-33.1)				
**Cardiovascular risk factors,%**															
Smoking	32.1	38.9	0.2	27.4	25.4	NA	63.2	64.8	0.17	34.8	31.1	0.618	57.1	54.5	NA
Hypertension	80.7	77.6	0.5	61.5	59	NA	60.4	63.2	< 0.01	53	48.5	0.637	56.5	61.1	NA
Hyperlipidemia	68.6	69.2	0.9	34.8	33.1	NA	56.5	58.6	0.05	45.5	40.8	0.633	39.7	57.9	NA
DM	38.5	35	0.5	34.6	30.3	NA	24.7	23.4	0.06	25.8	25.2	0.999	26.1	25	NA
Prior MI	32.9	45.6	0.001	15.8	17.7	NA	17.4	19.3	< 0.01	21.2	30.1	0.217	NA	NA	NA
Prior PCI	14.5	21.4	0.06	18.8	18.9	NA	15.1	17.4	< 0.01	16.7	22.3	0.434	NA	NA	NA
Prior CABG	NA	NA	NA	3.9	5.9	NA	5.1	9.9	< 0.01	6.1	4.9	0.738	NA	NA	NA
Heat failure	NA	NA	NA	NA	NA	NA	13.2	9.8	< 0.01	NA	NA	NA	NA	NA	NA
Stroke	12.9	6.6	0.05	6	8.5	NA	NA	NA	NA	NA	NA	NA	NA	NA	NA
Peripheral artery disease	17.2	18.1	0.8	11	12.6	NA	6.2	7.4	0.02	NA	NA	NA	NA	NA	NA
Chronic kidney disease	51.4	39.8	0.001	NA	NA	NA	0.8	0.9	> 0.99	NA	NA	NA	NA	NA	NA
Cardiac arrest	NA	NA	NA	NA	NA	NA	NA	NA	NA	NA	NA	NA	NA	NA	NA
**Hemodynamics and functional parameters**															
Mean heart rate, beats/min	NA	NA	NA	91	90	NA	NA	NA	NA	95.0 ± 20.0	98.0 ± 21.2	0.36	NA	NA	NA
				(72-107)	(73-109)										
SBP, mmHg	NA	NA	NA	100	100	NA	NA	NA	NA	82.0 ± 15.7	83.0 ± 21.2	0.742	NA	NA	NA
				(85-130)	(83-120)										
LV ejection fraction	NA	NA	NA	30 (21-40)	33 (25-40)	NA	NA	NA	NA	31.0 ± 9.6	30.3 ± 9.0	0.493	NA	NA	NA
**Angiographic parameters,%**															
Three-vessel disease	69.5	61.8	0.07	63.2	63.6	NA	NA	NA	NA	51.5	47.6	0.639	NA	NA	NA
TIMI-flow 3 post-PCI	NA	NA	NA	86.7	84.5	NA	NA	NA	NA	18.1	22.3	0.564	87	86	NA

*MV-PCI, multivessel percutaneous coronary intervention; CO-PCI, culprit vessel only percutaneous coronary intervention; BMI, body mass index; DM, diabetes mellitus; MI, myocardial infarction; PCI, percutaneous coronary intervention; CABG, coronary artery bypass grafting; SBP, systolic blood pressure; LV, left ventricular; TIMI, thrombolysis in myocardial infarction.*

### Efficacy Outcomes

**FIGURE 2:**
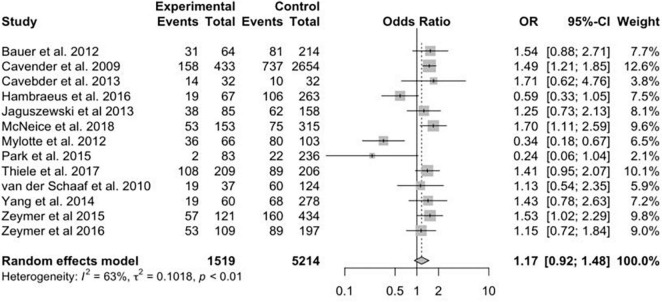
Short-term mortality with multivessel (MV) vs. culprit vessel-only (CO) percutaneous coronary intervention (PCI) in patients with ST-segment elevation myocardial infarction (STEMI) complicated by cardiogenic shock (CS).

**FIGURE 3:**
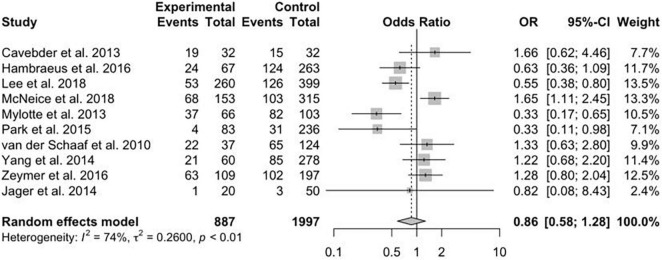
Long-term mortality with multivessel (MV) vs. culprit vessel-only (CO) percutaneous coronary intervention (PCI) in patients with ST-segment elevation myocardial infarction (STEMI) complicated by cardiogenic shock (CS).

**FIGURE 4:**
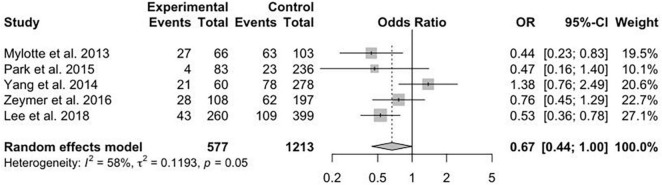
Cardiac death with multivessel (MV) vs. culprit vessel-only (CO) percutaneous coronary intervention (PCI) in patients with ST-segment elevation myocardial infarction (STEMI) complicated by cardiogenic shock (CS).

**FIGURE 5:**
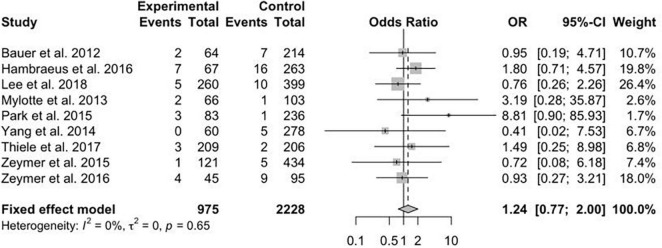
Myocardial reinfarction (MI) with multivessel (MV) vs. culprit vessel-only (CO) percutaneous coronary intervention (PCI) in patients with ST-segment elevation myocardial infarction (STEMI) complicated by cardiogenic shock (CS).

**FIGURE 6:**
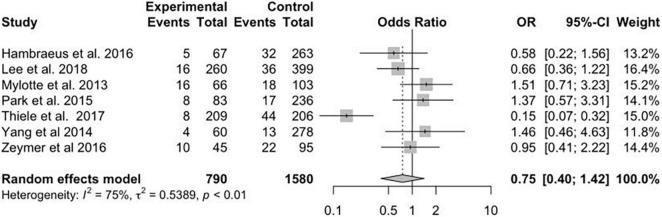
Repeat revascularization reinfarction (MI) with multivessel (MV) vs. culprit vessel-only (CO) percutaneous coronary intervention (PCI) in patients with ST-segment elevation myocardial infarction (STEMI) complicated by cardiogenic shock (CS).

A total of 13 studies reported the data on primary efficacy endpoint of short-term all-cause mortality. There was no statistically significant difference in short-term mortality with MV-PCI compared with CO-PCI (OR = 1.17, 95% CI = 0.92–1.48, *p* < 0.01, *I*^2^ = 63%; Figure 2). We found that there might be publication bias for all-cause short-term mortality (Supplementary Figure 1; Egger’s test: *p* = 0.006). There were 10 studies reporting the outcome of long-term mortality and 5 studies reporting cardiac death. No significant difference in long-term mortality (OR = 0.86, 95% CI = 0.58–1.28, *p* < 0.01, *I*^2^ = 74%; Figure 3) and a borderline significant difference in cardiac death (OR = 0.67, 95% CI = 0.44–1.00, *p* = 0.23, *I*^2^ = 60%; Figure 4) were found between MV-PCI and CO-PCI groups. Furthermore, there might be no publication bias for long-term mortality (Supplementary Figure 2; Egger’s test: *p* = 0.860) and myocardial reinfarction (Supplementary Figure 3; Egger’s test: *p* = 0.991). Data on repeat revascularization and myocardial reinfarction were reported in 7 and 9 studies, respectively. No statistically significant difference in the risk of myocardial reinfarction (OR = 1.24, 95% CI = 0.77–2.00, *p* = 0.65, *I*^2^ = 0%; Figure 5) or repeat revascularization (OR = 0.75, 95% CI = 0.40–1.42, *p* < 0.01, *I*^2^ = 75%; Figure 6) was found between the two groups. There was publication bias for the secondary efficacy endpoints of repeat revascularization (Supplementary Figure 4; Egger’s test: *p* = 0.532) and cardiac death (Supplementary Figure 5; Egger’s test: *p* = 0.587).

Sensitivity analyses were performed for the efficacy outcomes to assess the robustness of result using the leave-one-out method. It was demonstrated that excluding a single study did not significantly alter the overall outcomes for long-term mortality, cardiac death, myocardial reinfarction, and repeat revascularization. However, for short-term mortality, excluding the study of Mylotte et al. (20) changed the statistical significance of the overall pooled estimate (OR = 1.31; 95% CI, 1.10–1.57; Supplementary Figures 6–10).

### Safety Outcomes

**FIGURE 7:**
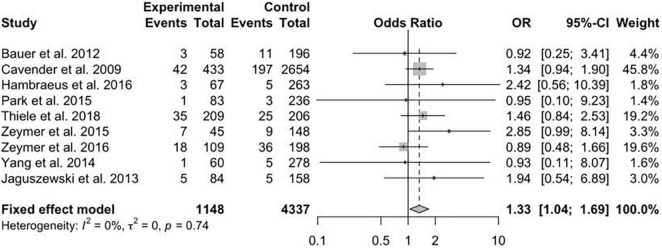
Renal failure with multivessel (MV) vs. culprit vessel-only (CO) percutaneous coronary intervention (PCI) in patients with ST-segment elevation myocardial infarction (STEMI) complicated by cardiogenic shock (CS).

**FIGURE 8:**
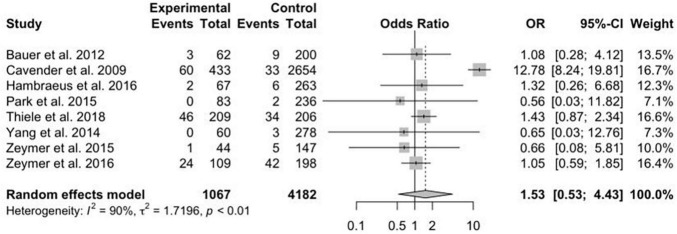
Bleeding with multivessel (MV) vs. culprit vessel-only (CO) percutaneous coronary intervention (PCI) in patients with ST-segment elevation myocardial infarction (STEMI) complicated by cardiogenic shock (CS).

**FIGURE 9:**
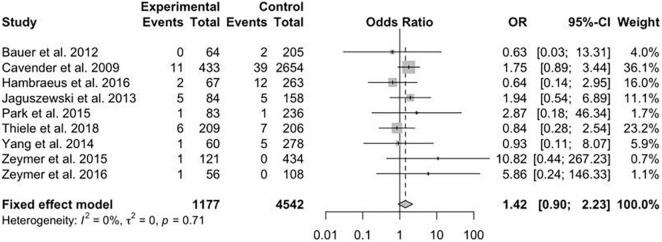
Stroke with multivessel (MV) vs. culprit vessel-only (CO) percutaneous coronary intervention (PCI) in patients with ST-segment elevation myocardial infarction (STEMI) complicated by cardiogenic shock (CS).

The data concerning renal failure has been reported in 9 studies. There was a statistically significant higher risk of acute renal failure in patients treated with MV-PCI compared with CO-PCI (OR = 1.33; 95% CI, 1.04–1.69; *p* = 0.74, *I*^2^ = 0%; Figure 7). The safety outcome of bleeding was reported in 8 studies, and no statistically significant difference was found between MV-PCI and CO-PCI (OR = 1.53; 95% CI, 0.53–4.43; *p* < 0.01, *I*^2^ = 90%; Figure 8). Finally, stroke was reported in 9 studies, and no significant difference was found between the two groups (OR = 1.42; 95% CI, 0.90–2.23; *p* = 0.71, *I*^2^ = 0%; Figure 9). Publication bias was observed for the safety outcome of bleeding (Supplementary Figure 11; Egger’s test: *p* = 0.069), but no publication bias was found about renal failure (Supplementary Figure 12; Egger’s test: *p* = 0.281) and stroke (Supplementary Figure 13; Egger’s test: *p* = 0.587).

Sensitivity analyses were performed on the safety outcomes of renal failure, bleeding, and stroke to assess the robustness of results. Excluding a single study did not significantly alter the overall results of bleeding and stroke. However, for renal failure, excluding the study of Cavender et al. (14) and Thiele et al. (31) affects the statistical significance of the overall pooled estimates (OR = 1.32; 95% CI, 0.95–1.57 and OR = 1.30; 95% CI, 0.99–1.70; Supplementary Figures 14–16).

## Discussion

This meta-analysis including 15 studies has suggested that there is no significant difference in the efficiency outcomes of short/long-term mortality, revascularization, myocardial reinfarction, and safety outcomes of bleeding and stroke in patients with STEMI complicated by CS treated with MV-PCI compared to CO-PCI. Our results were supported by previous meta-analysis (32), which demonstrated that there was no statistical difference of any efficiency or safety outcomes between the two groups, although they focused on patients with AMI with CS. Our study also revealed that MV-PCI could increase the risk of acute renal failure, and this result has been supported by the newly published study (33). However, another meta-analysis (34) indicated that the incidence of MI and revascularization were significantly reduced, but all-cause death was not affected by the revascularization strategy. Previous meta-analyses concerning this topic have reported inconsistent results. The study of Kolte et al. (35) showed that there was no difference in short/long-term outcomes in the two groups, whereas another study (36) reported higher short-term mortality in the MV-PCI treatment group. However, the two studies did not include RCTs data, and the second study included all patients with AMI without separating patients with STEMI and NSTEMI.

At present, it is still a controversial issue about the optimal revascularization strategy in patients with STEMI complicated by CS. Theoretically speaking, MV-PCI of non-culprit arteries may improve survival rate of patients with AMI through limiting infarct size and preserving left ventricular function. However, in our study, immediate MV-PCI did not decrease the short/long-term mortality in patients with STEMI complicated by CS, compared with CO-PCI. The non-statistical difference may be due to the difference of baseline characteristics between the two groups and inability to adjust for patient and operator characteristics, as almost all the included studies were non-randomized. And patients who underwent MV-PCI were sicker and were prone to have adverse outcomes, which could counteract the potential benefit of MV-PCI. Nevertheless, two previous trials reported that there was no difference in all-cause mortality among patients with STEMI without CS between MV-PCI and CO-PCI treatment groups (10, 11). During our search process of study selection, few studies were found to evaluate the efficacy and safety of CO-PCI compared to MV-PCI treatment among patients with AMI. However, these studies focused on different populations [AMI and CS (32, 36–38); AMI, CS, and MVD (39); and STEMI and MVD (40)], thus gave different conclusions and could not provide optimal strategies for patients with STEMI with CS. In addition, the location of culprit lesion may be one of the sources of heterogeneity in mortality. Lee et al. (25) reported that compared with IRA-only PCI group, the all-cause mortality of indicated culprit in the MVP group decreased [left main or left anterior descending (LAD) culprit: hazard ratio (HR) = 0.53 (0.36–0.77); left circumflex artery (LCX) or right coronary artery (RCA) culprit: HR = 0.57 (0.32–1.02)]. However, Jaguszewski et al. (18) found that when left main is the diseased vessel, there was no difference in the in-hospital mortality between single-vessel PCI group and multivessel PCI group. More data from RCTs with large sample are needed to investigate the relationship between the location of culprit lesions and mortality.

Both RCTs and meta-analyses of RCTs have shown that there is a significant reduction in repeat revascularization in patients with STEMI without CS with MV-PCI when compared with CO-PCI (11, 41, 42). This may be because complete revascularization leads to subsequent improved ventricular function and a lower subsequent incidence of heart failure (20, 32). However, no significant difference in reinfarction or repeat revascularization was found in patients with STEMI with CS in the two groups, and this result was also supported by previous meta-analysis, which focused on patients with STEMI with CS (34). There is a fact that 100% of patients who were treated with MV-PCI would undergo additional revascularization of the non-infarct-related arteries upfront, and this may influence the endpoint of repeat revascularization. In patients with STEMI without CS, MV-PCI did not increase the risks of bleeding and stroke (9–11, 41). For patients aged 75 years and older with MI (either STE or NSTE), functionally guided complete revascularization may reduce the occurrence of the composite patient-oriented endpoint of all-cause death, MI, stroke, and ischemia-driven revascularization (42). On the contrary, we found higher rates of renal failure with MV-PCI compared with CO-PCI in patients with STEMI with CS. As reported, presentation with STEMI and CS is associated with 2- to 3-fold higher risk of developing acute kidney injury after PCI (43). In addition, Park et al. (21) found the risk of contrast-induced nephropathy was 9.08 times higher in the MV-PCI group than in the CO-PCI group. This together with the use of higher amounts of contrast during MV-PCI may explain the statistically association between MV-PCI and acute renal failure compared with CO-PCI, but there is a lack of data for analyzing the problem quantitatively.

Finally, some limitations should be acknowledged in this study. First, we only included one RCT study in the meta-analysis, further high-quality RCTs, which decrease the selection bias and unmeasured confounding maximally, are needed to support our results. Second, heterogeneity across studies raise from the different entry criteria, study population, and follow-up time that limit the conclusions’ generalization. Finally, the definition of efficiency and safety outcomes varied among included studies, which could also introduce heterogeneity.

## Conclusion

This study suggests that there is no additional benefit in either efficiency outcomes of short/long-term mortality, revascularization, myocardial reinfarction, or safety outcomes of bleeding and stroke in patients with STMEI complicated by CS treated with MV-PCI, compared to CO-PCI. In addition, MV-PCI may increase the risk of acute renal failure. Nonetheless, further RCTs with high quality are needed in the real world to provide optimal revascularization strategy.

## Data Availability Statement

The original contributions presented in the study are included in the article/Supplementary Material, further inquiries can be directed to the corresponding author/s.

## Author Contributions

BX and HY provided the idea and wrote this manuscript. YZ, WY, and YH verified the analytical methods. QS encouraged all authors to finish this report. All authors contributed to the article and approved the submitted version.

## Conflict of Interest

The authors declare that the research was conducted in the absence of any commercial or financial relationships that could be construed as a potential conflict of interest. The reviewer YL declared a past collaboration with one of the authors QS to the handling editor.

## Publisher’s Note

All claims expressed in this article are solely those of the authors and do not necessarily represent those of their affiliated organizations, or those of the publisher, the editors and the reviewers. Any product that may be evaluated in this article, or claim that may be made by its manufacturer, is not guaranteed or endorsed by the publisher.
