# Supplementary material for: Multivessel vs. Culprit Vessel-Only Percutaneous Coronary Intervention for ST-Segment Elevation Myocardial Infarction in Patients With Cardiogenic Shock: An Updated Systematic Review and Meta-Analysis
Source: Front Cardiovasc Med. 2022 Apr 15;9:735636. doi: 10.3389/fcvm.2022.735636 (PMC9051032; doi:10.3389/fcvm.2022.735636)

**Table S1.** The quality assessment of included studies.

| Study | Study type | Selection | Comparability | Outcome/Exposure |
| --- | --- | --- | --- | --- |
| Cavender, 2009 | Observational | *** | * | *** |
| Van der schaaf, 2010 | Observational | *** | * | *** |
| Bauer, 2012 | Observational | *** | * | *** |
| Cavender, 2013 | Observational | *** | * | *** |
| Yang, 2013 | Observational | *** | * | *** |
| Mylotte, 2013 | Observational | *** | * | *** |
| Zeymer, 2014 | Observational | *** | * | *** |
| Park, 2015 | Observational | *** | * | *** |
| Zeymer, 2016 | Observational | *** | * | *** |
| Jager, 2014 | Observational | *** | * | *** |
| McNeice, 2018 | Observational | *** | * | *** |
| Lee, 2018 | Observational | *** | * | *** |
| Jaguszawski, 2013 | Observational | *** | * | ** |
| Hambraeus, 2016 | Observational | *** | * | *** |
| Thiele, 2017 | RCT | NA | NA | NA |

**Figure S1.** Funnel plot for short-term mortality. Comparing the odds ratio (x axis) of individual studies to standard error of odds ratio (y-axis) on logarithmic scale. Visual inspection shows the studies are not evenly distributed around no effect line, thus favoring exist of selection bias.

**
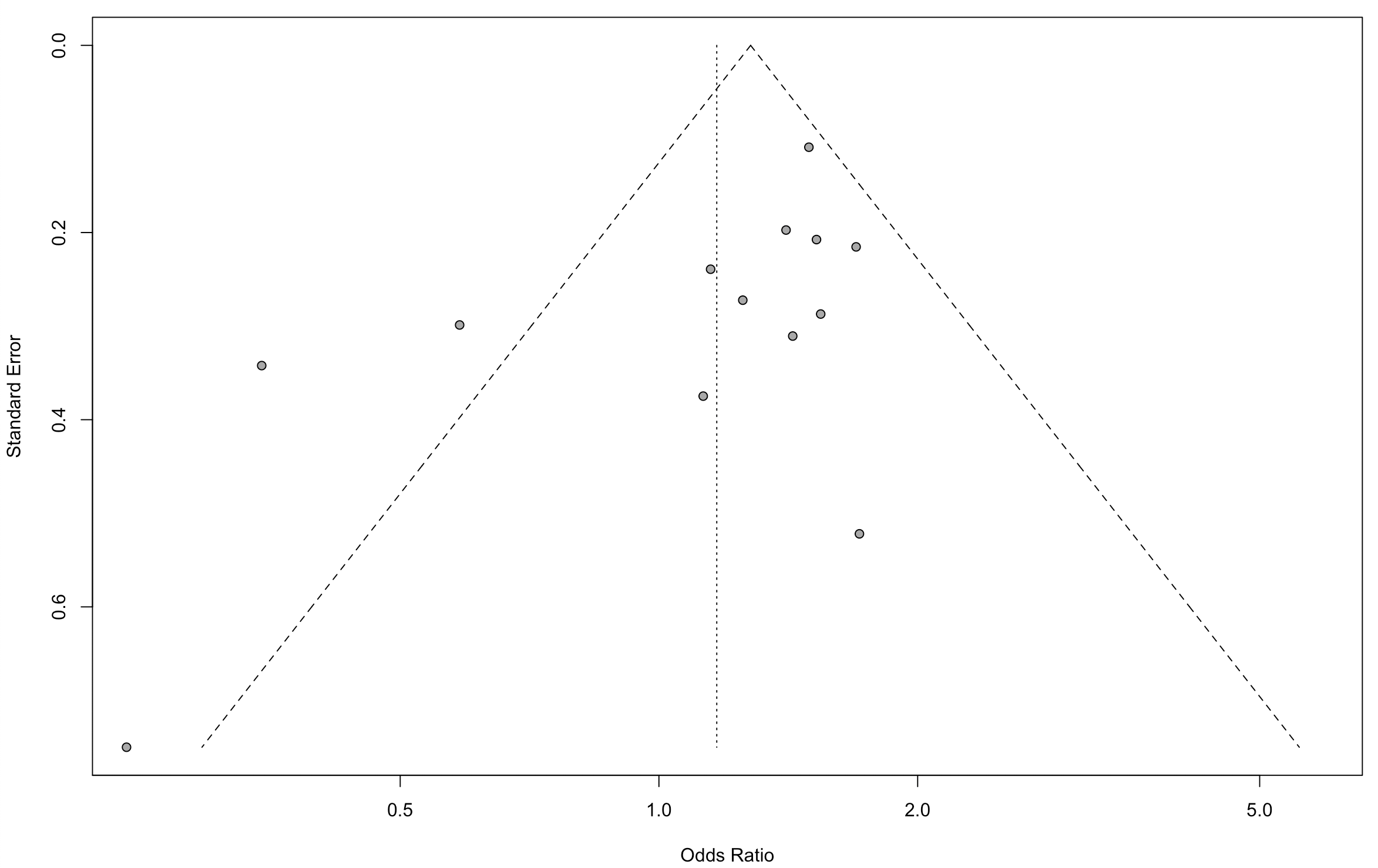
**

**Figure S2.** Funnel plot for long-term mortality. Comparing the odds ratio (x axis) of individual studies to standard error of odds ratio (y-axis) on logarithmic scale. Visual inspection shows the studies are evenly distributed around no effect line, thus favoring lack of selection bias.


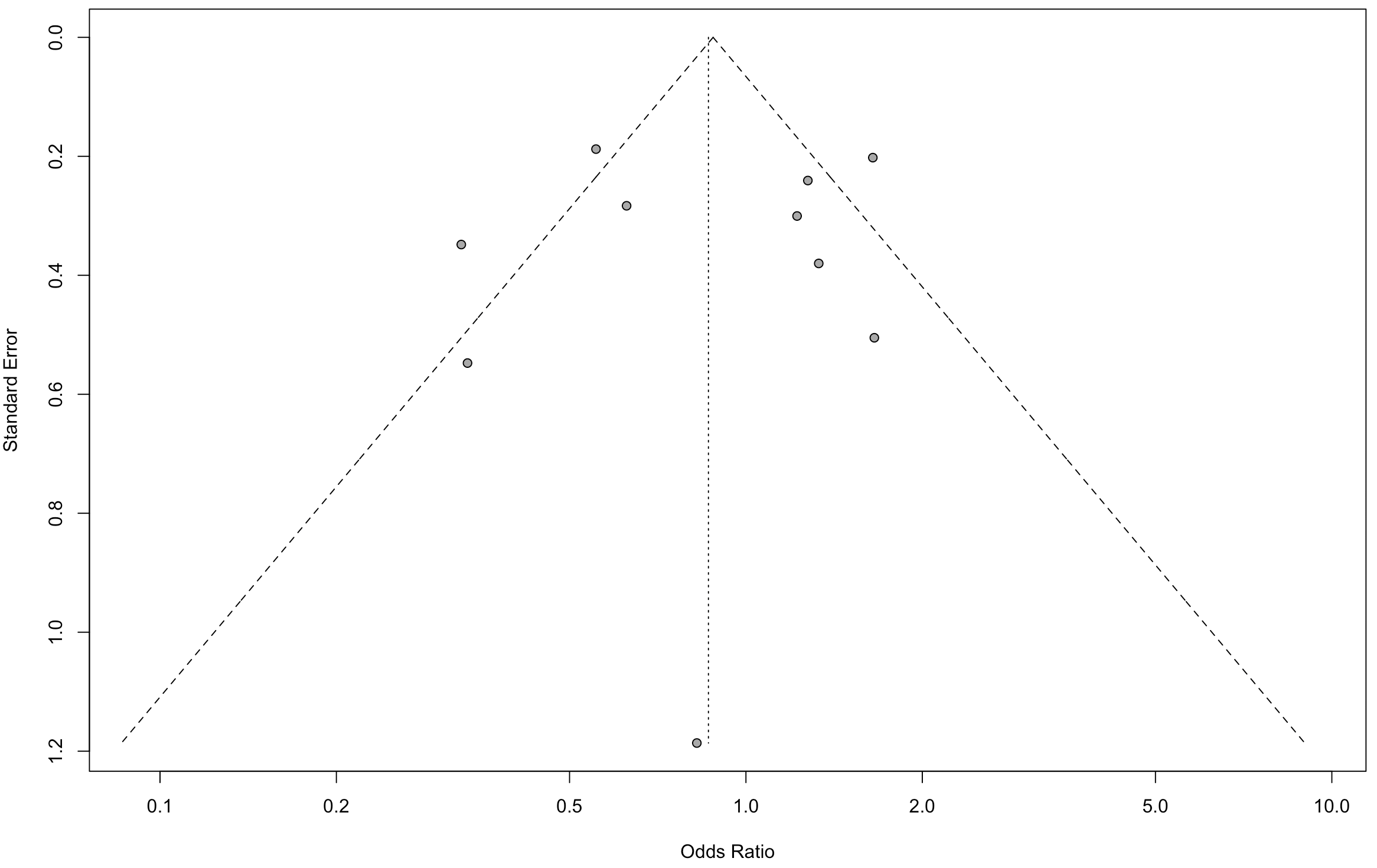


**Figure S3.** Funnel plot for myocardial reinfarction. Comparing the odds ratio (x axis) of individual studies to standard error of odds ratio (y-axis) on logarithmic scale. Visual inspection shows the studies are evenly distributed around no effect line, thus favoring lack of selection bias.

**
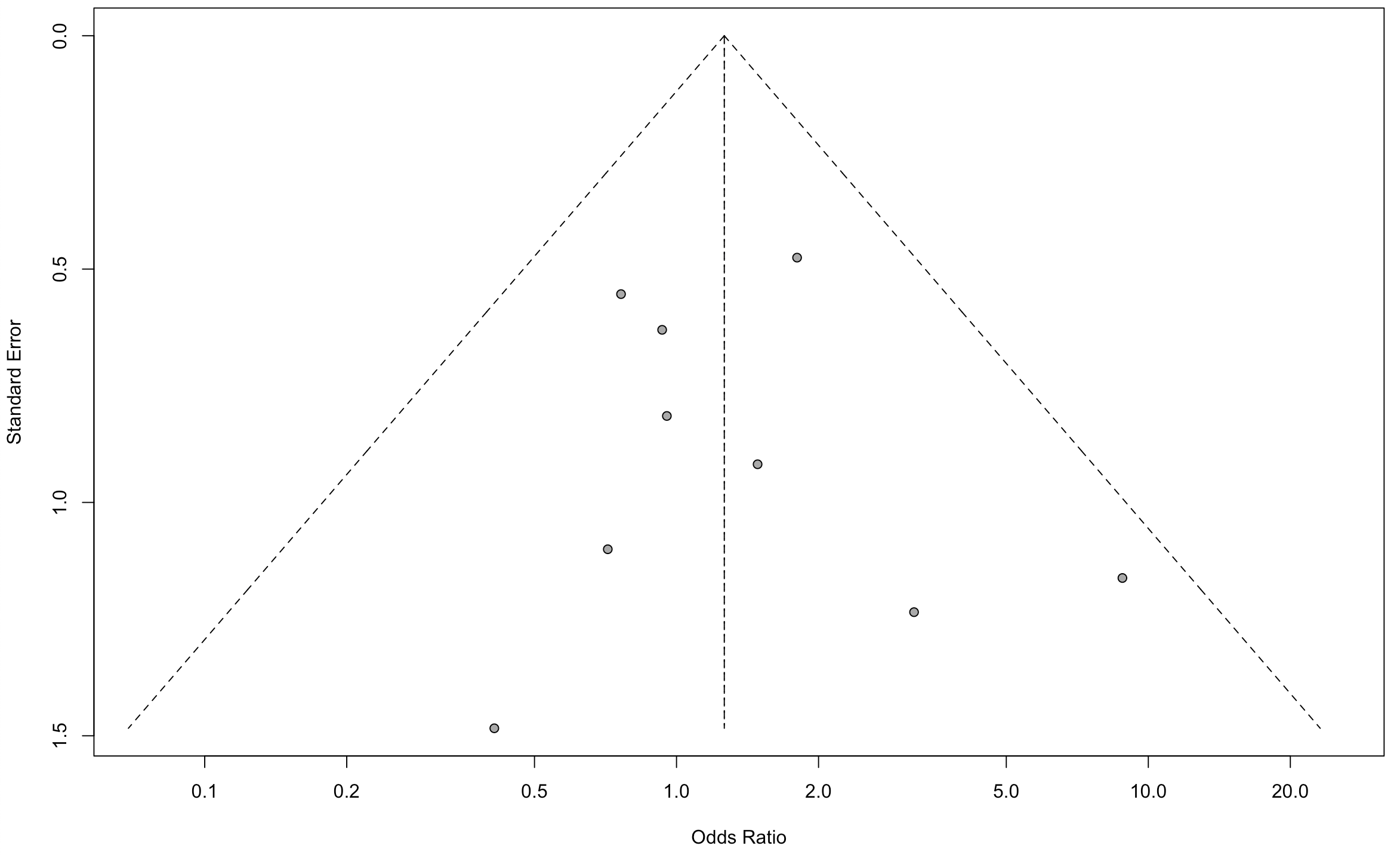
**

**Figure S4.** Funnel plot for repeat revascularization. Comparing the odds ratio (x axis) of individual studies to standard error of odds ratio (y-axis) on logarithmic scale. Visual inspection shows the studies are not evenly distributed around no effect line, thus favoring exist of selection bias.


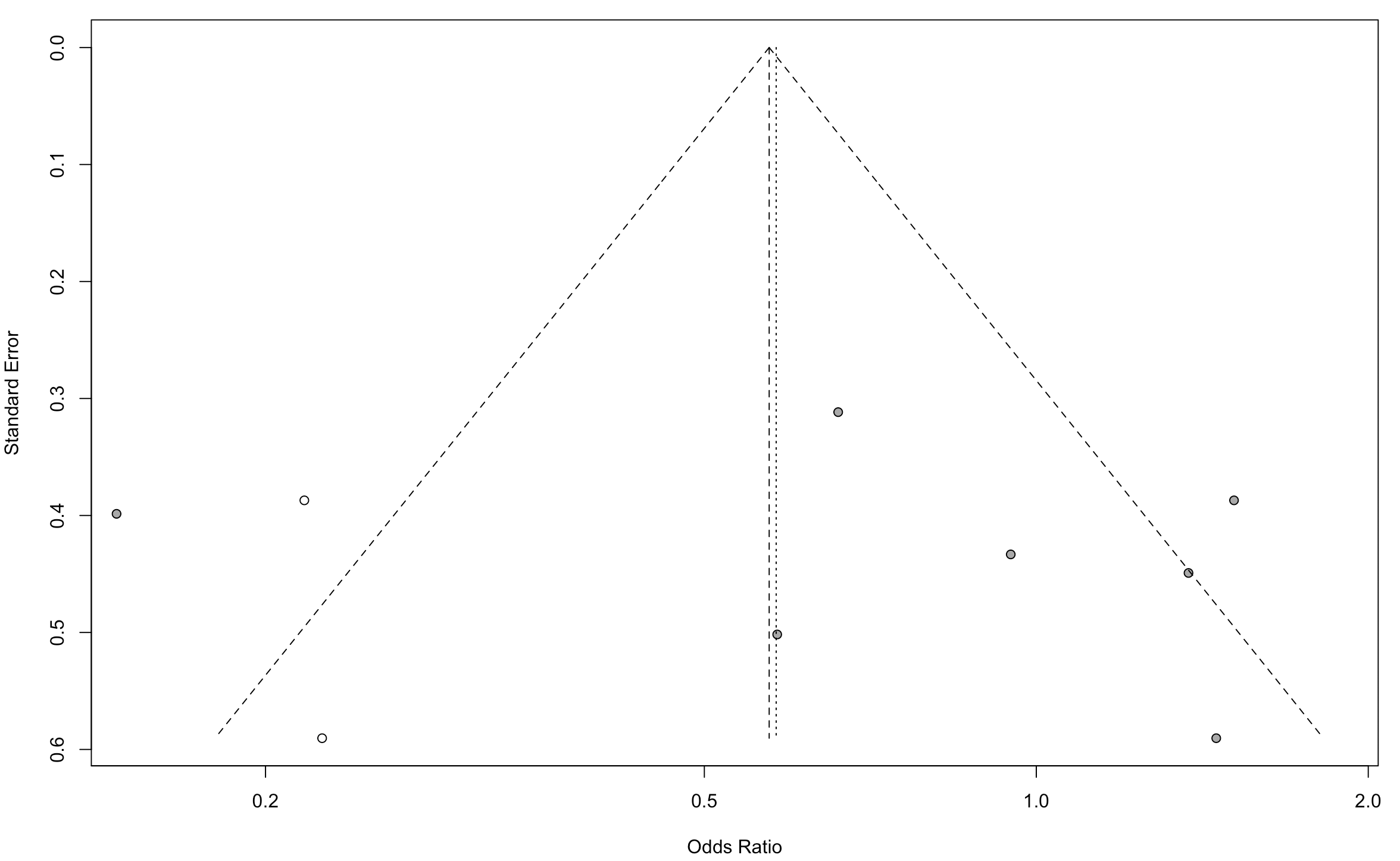


**Figure S5.** Funnel plot for cardiac death. Comparing the odds ratio (x axis) of individual studies to standard error of odds ratio (y-axis) on logarithmic scale. Visual inspection shows the studies are not evenly distributed around no effect line, thus favoring exist of selection bias.


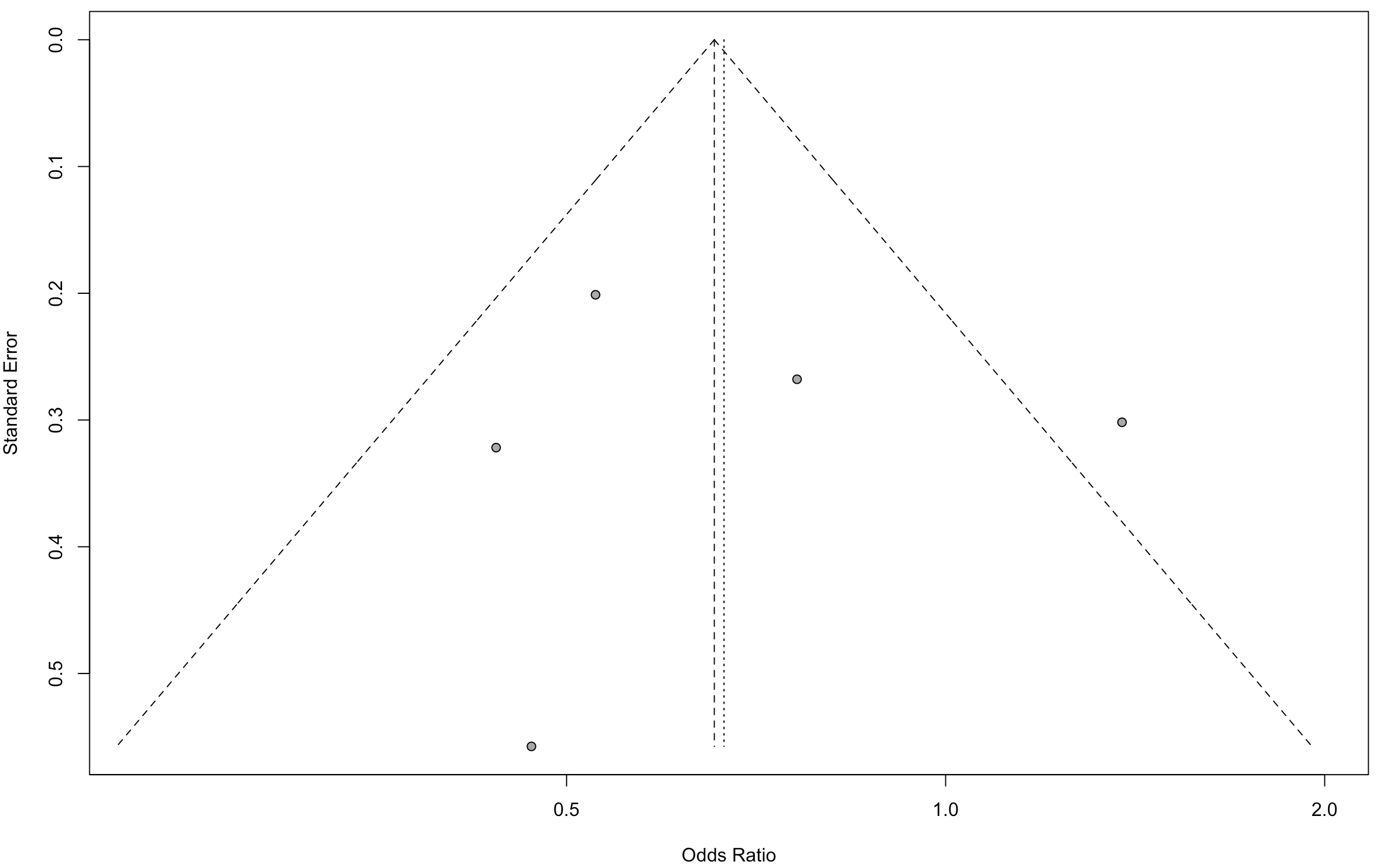


**Figure S6.** Sensitivity analysis for short-term mortality.

**
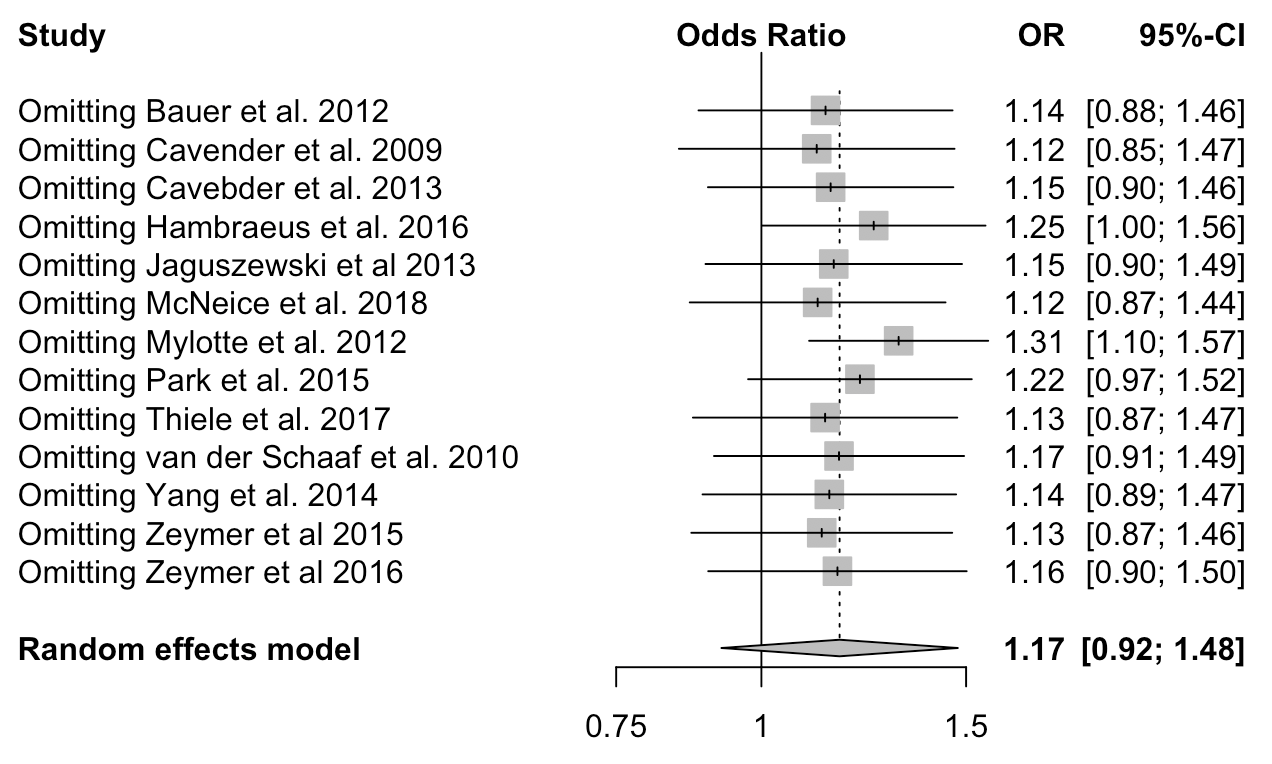
$**

**Figure S7.** Sensitivity analysis for long-term mortality.

**
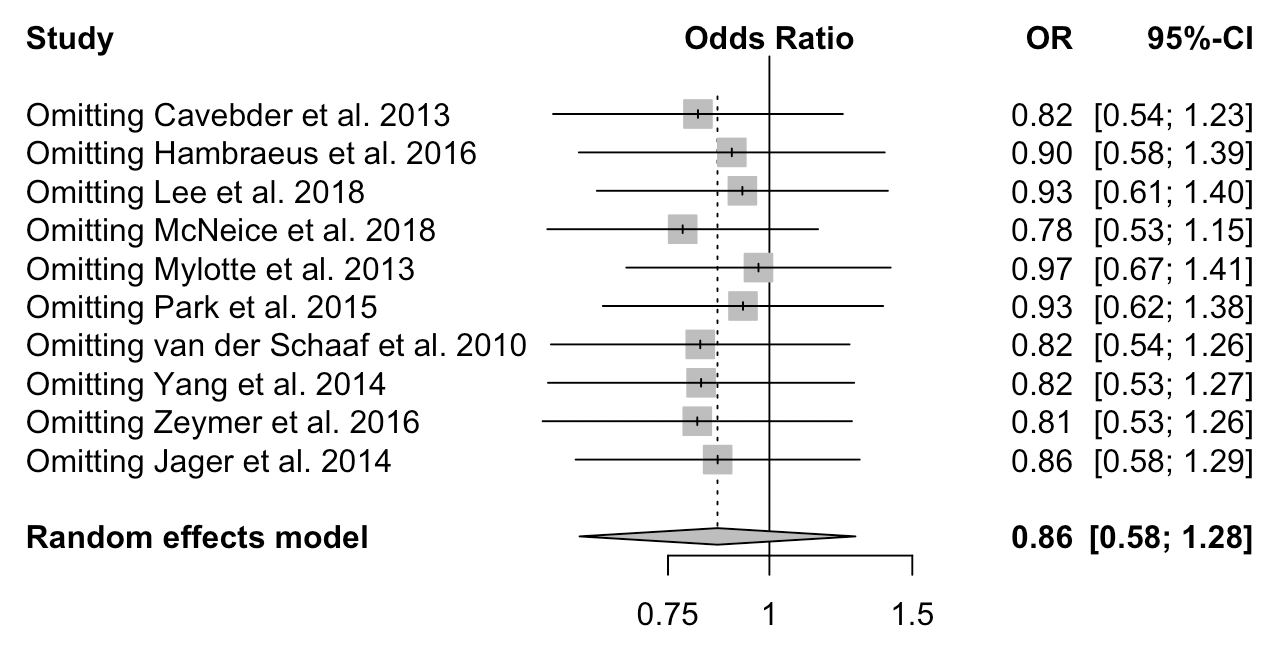
**

**Figure S8.** Sensitivity analysis for cardiac death.


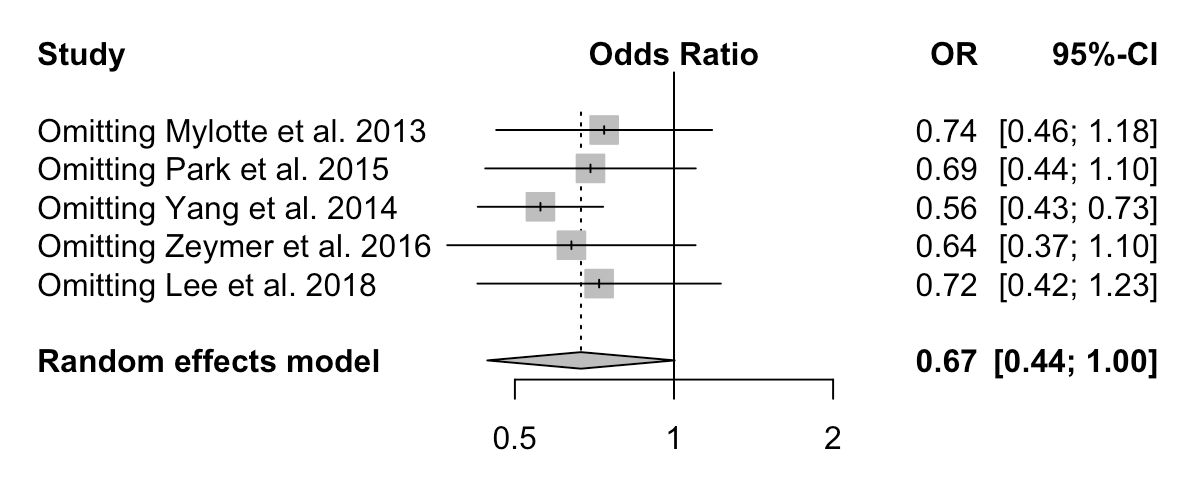


**Figure S9.** Sensitivity analysis for myocardial reinfarction.


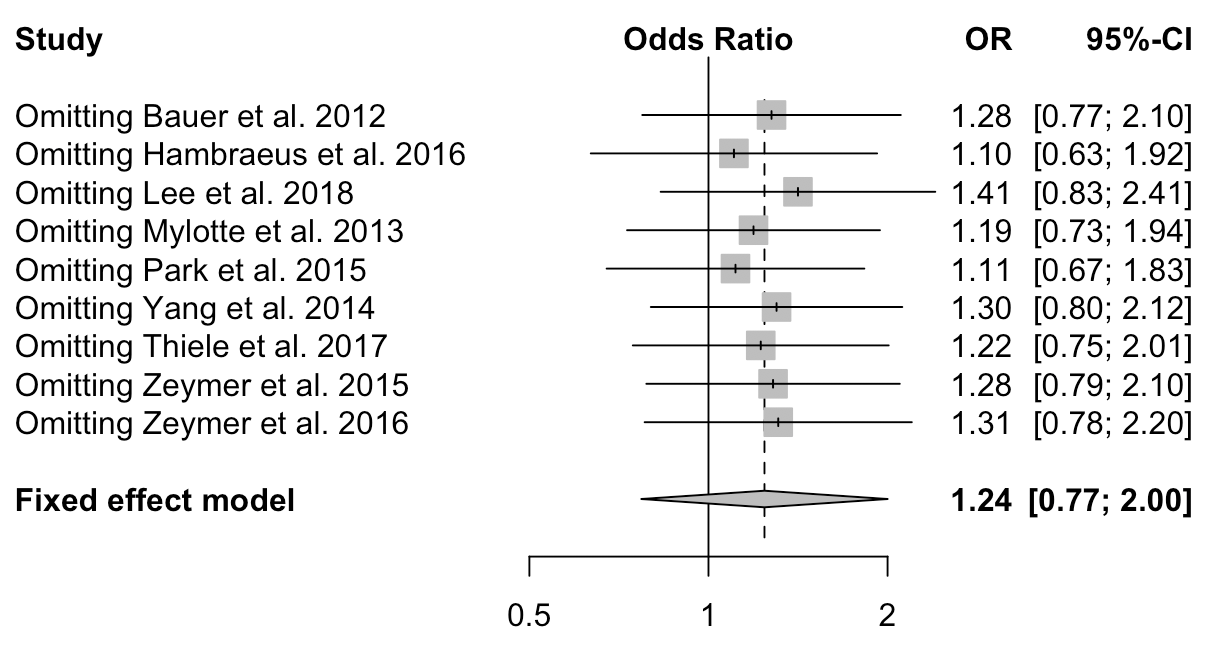


**Figure S10.** Sensitivity analysis for repeat revascularization.


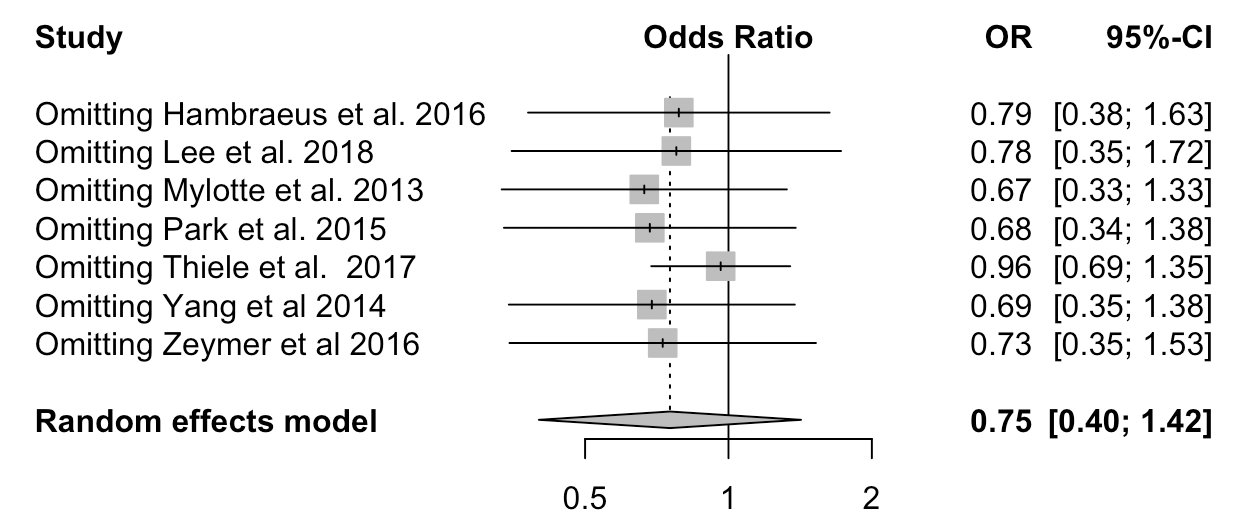


**Figure S11.** Funnel plot for bleeding. Comparing the odds ratio (x axis) of individual studies to standard error of odds ratio (y-axis) on logarithmic scale. Visual inspection shows the studies are not evenly distributed around no effect line, thus favoring exist of selection bias.


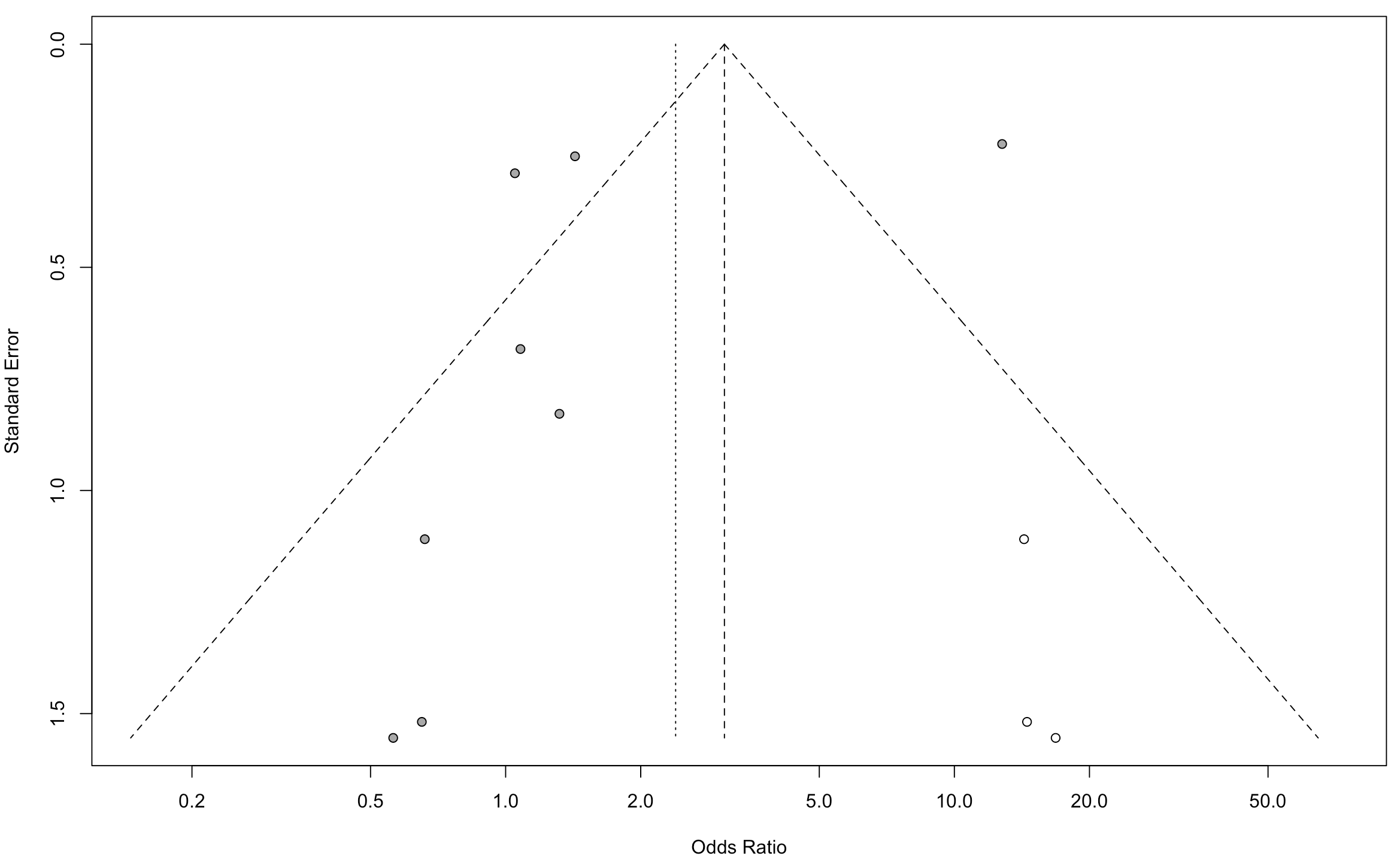


**Figure S12.** Funnel plot for renal failure. Comparing the odds ratio (x axis) of individual studies to standard error of odds ratio (y-axis) on logarithmic scale. Visual inspection shows the studies are evenly distributed around no effect line, thus favoring lack of selection bias.
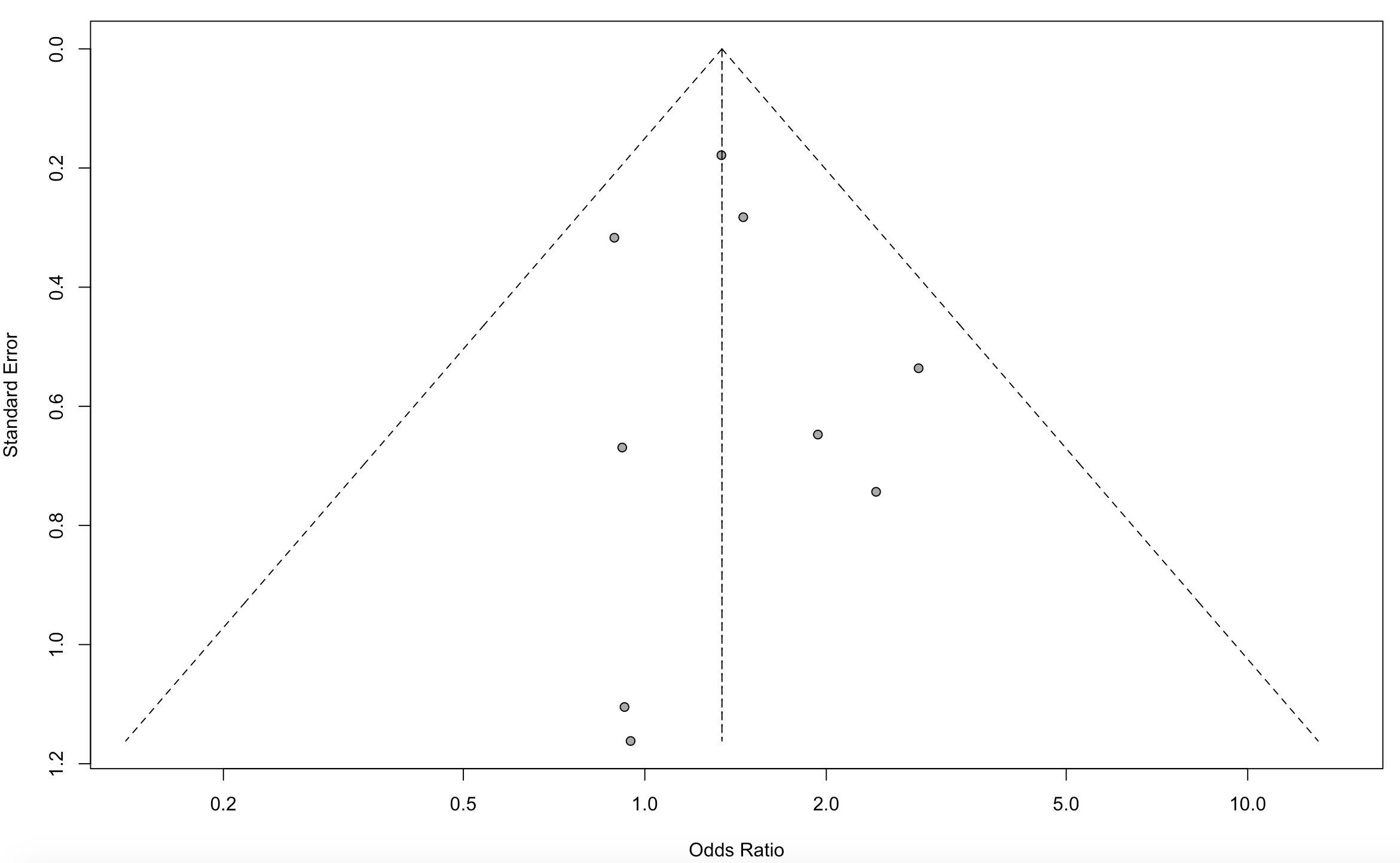


**Figure S13.** Funnel plot for stroke. Comparing the odds ratio (x axis) of individual studies to standard error of odds ratio (y-axis) on logarithmic scale. Visual inspection shows the studies are evenly distributed around no effect line, thus favoring lack of selection bia**
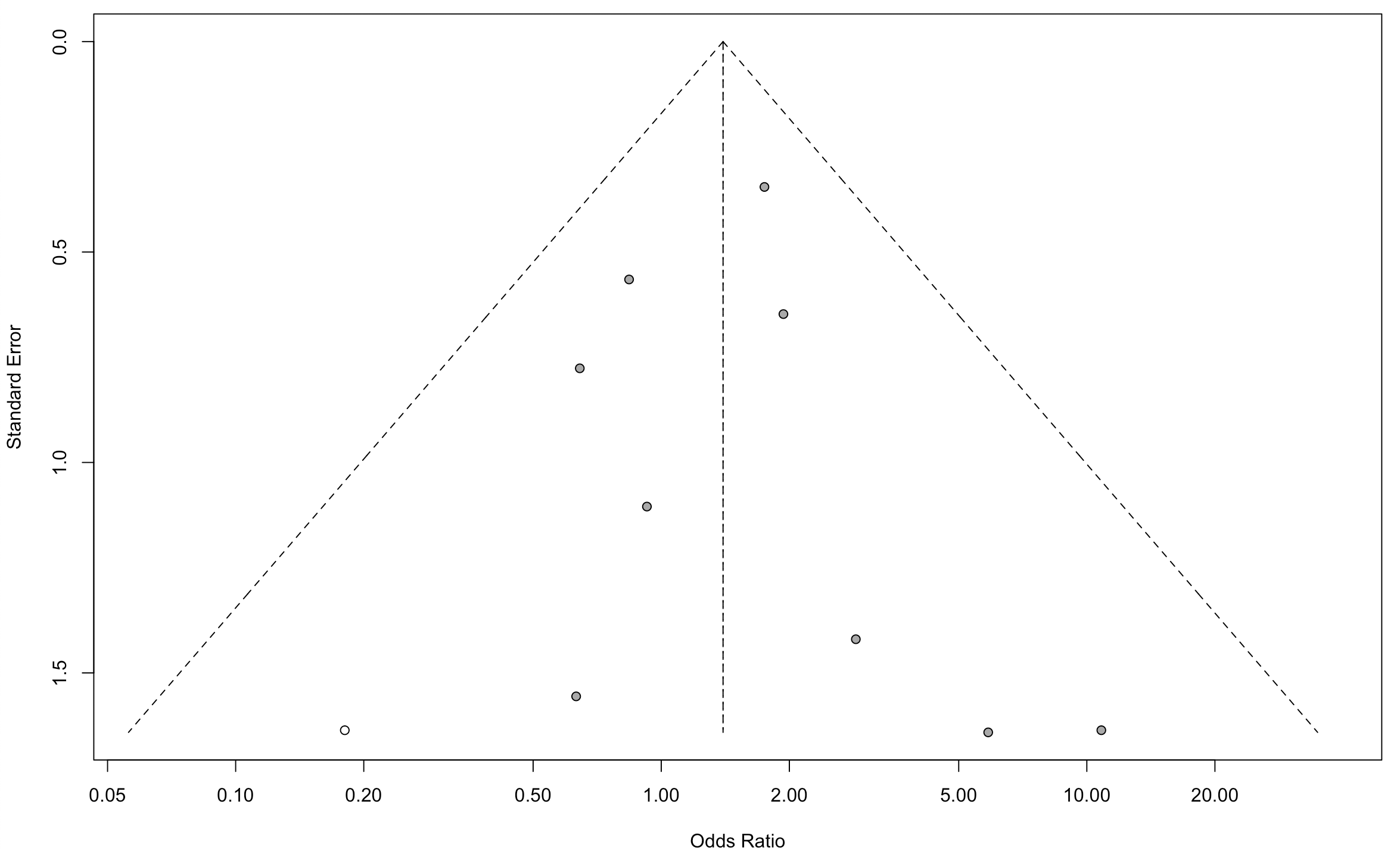
、**

**Figure S14.** Sensitivity analysis for renal failure.


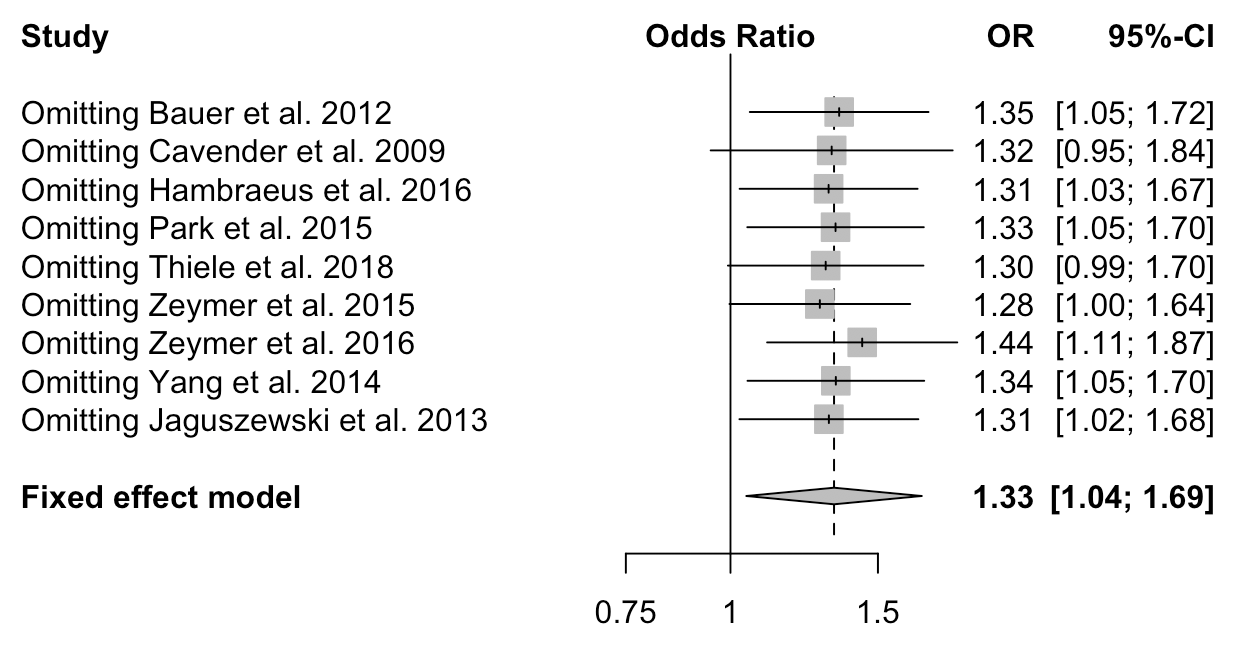


**Figure S15.** Sensitivity analysis for bleeding.


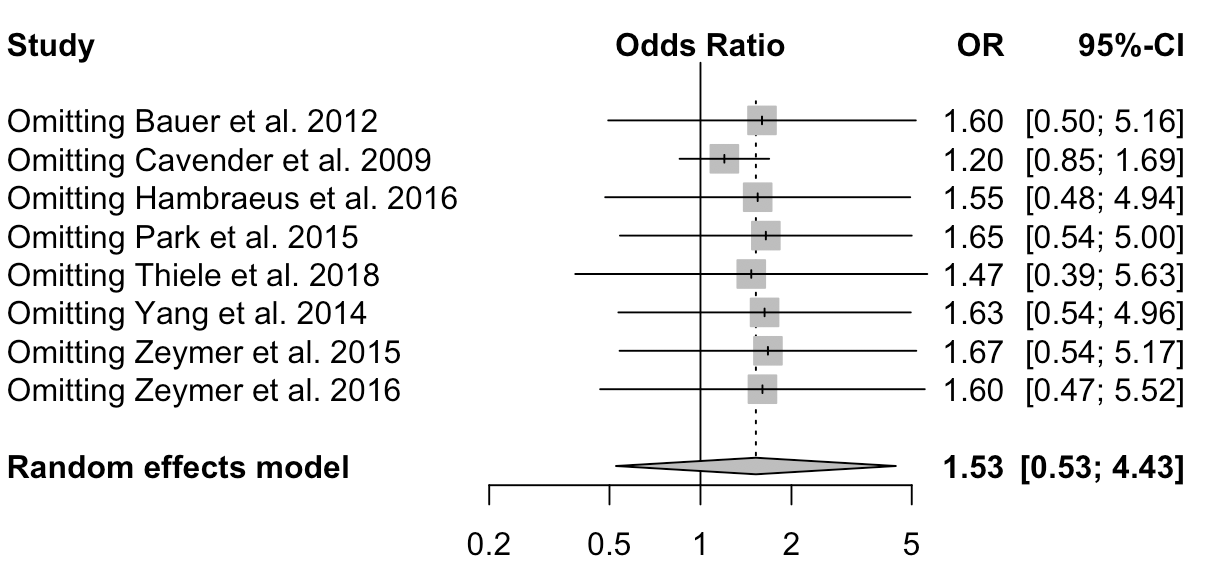


**Figure S16.** Sensitivity analysis for stroke.


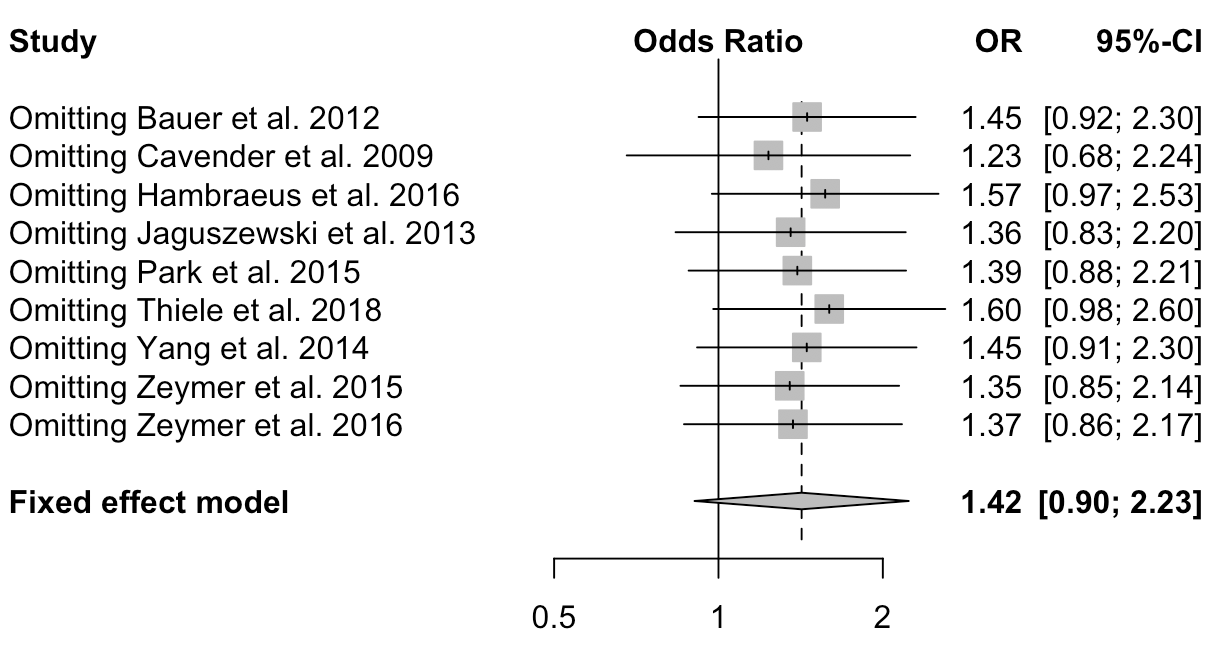

Supplement: Supplementary file 2 [file Data_Sheet_2.docx]
